# Supplementary material for: eIF4A supports an oncogenic translation program in pancreatic ductal adenocarcinoma
Source: Nat Commun. 2019 Nov 13;10:5151. doi: 10.1038/s41467-019-13086-5 (PMC6853918; doi:10.1038/s41467-019-13086-5)
Supplement: Supplementary file 2 — Description of Additional Supplementary Files [file 41467_2019_13086_MOESM2_ESM.docx]

Description of Additional Supplementary Files

**Supplementary Data 1**

List of transcripts that are translationally up or down regulated upon CR-31 treated in normal (N) organoids (n = 3). apvSlope = common slope of the two treatment groups; apvEff = log2 fold-change; apvRvmP = p-values; apvRvmPAdj = Adjusted p-values.

**Supplementary Data 2**

List of transcripts that are translationally up or down regulated upon CR-31 treated in *Kras^G12D^;p53^R172H^* (KP) organoids (n = 3). apvSlope = common slope of the two treatment groups; apvEff = log2 fold-change; apvRvmP = p-values; apvRvmPAdj = Adjusted p-values.

**Supplementary Data 3**

List of transcripts that are translationally up or down regulated in *Kras^G12D^;p53^R172H^* (KP) vs normal N organoids (n = 3). apvSlope = common slope of the two treatment groups; apvEff = log2 fold-change; apvRvmP = p-values; apvRvmPAdj = Adjusted p-values.
