# Supplementary material for: eIF4A supports an oncogenic translation program in pancreatic ductal adenocarcinoma
Source: Nat Commun. 2019 Nov 13;10:5151. doi: 10.1038/s41467-019-13086-5 (PMC6853918; doi:10.1038/s41467-019-13086-5)
Supplement: Supplementary file 1 — Supplementary Information [file 41467_2019_13086_MOESM1_ESM.pdf]

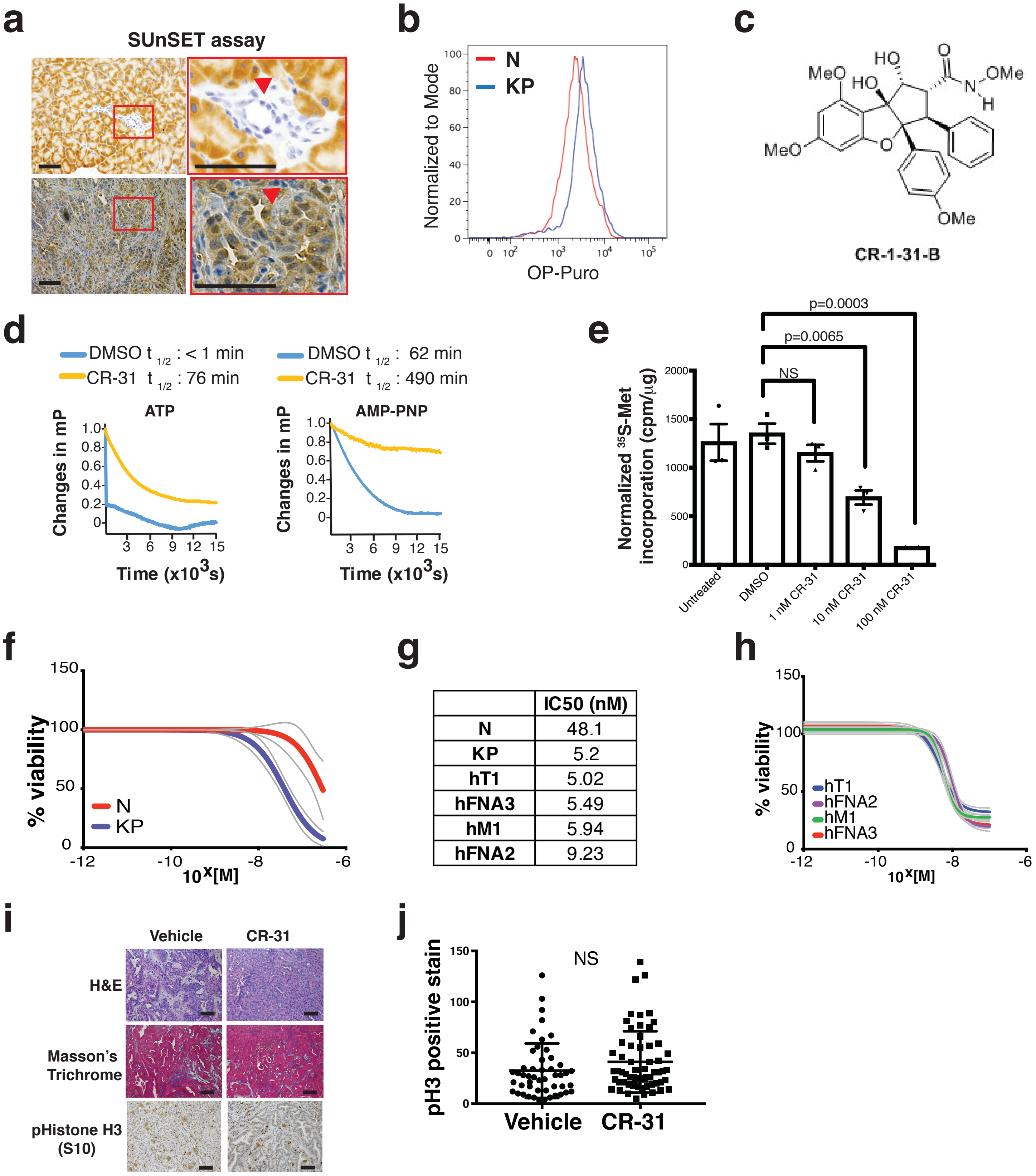


| | IC50 (nM) |
|---|---|
| N | 48.1 |
| KP | 5.2 |
| hT1 | 5.02 |
| hFNA3 | 5.49 |
| hM1 | 5.94 |
| hFNA2 | 9.23 |

# Supplementary Figure 1

**Supplementary Figure 1. eIF4A is an effective therapeutic target in PDA**

a. *In vivo* protein synthesis (quantified by SUnSET) in ductal epithelial cells of normal pancreas (top) and adenocarcinoma (bottom) in mice 30 min following intraperitoneal puromycin (40 nmol $g^{-1}$ body weight) injection. Red insets of each image enlarged on right hand side. Red arrows point to ducts in normal pancreas (top) and ductal tumour glands in pancreatic adenocarcinoma (bottom). Scale bars, 50 μm.
b. Normal (N), and $Kras^{G12D};p53^{R172H}$ (KP) organoids were incubated with 10 μM O-Propargyl-Puromycin (OP-Puro) for 30 min and then analyzed by flow cytometry to determine the level of OP-Puro conjugation with polypeptide chains. Representative data from 3 biological replicates.
c. Chemical structure of CR-1-31-B (CR-31).
d. Binding of eIF4A1 to FAM-labelled $(AG)_{10}$ RNA and measurements of off rates with unlabelled RNA using fluorescent polymerization. mP, millipolarization unit. Data are mean +/– S.D., n = 4.
e. [$^{35}$S]-Met incorporation into protein from KP organoids treated with increasing concentrations of CR-31 for 1 h. Data are mean +/– S.D., Student's *t*-test, n = 3. NS = not significant.
f. Cell viability of N and KP organoids treated with increasing concentrations of CR-31 for 72 h measured by area covered using CellInSight CX5. Dotted lines indicate 95% confidence intervals, n = 5.
g. $IC_{50}$ values of CR-31 in murine organoids (N and KP) and patient-derived PDA cell lines (hT1, hFNA2, hFNA3 and hM1).
h. Cell viability of a panel of patient-derived PDA cell lines upon 72 h of treatment with increasing concentrations of CR-31. Dotted lines, 95% confidence intervals, n = 6.
i. Hematoxylin and Eosin (H&E), Masson's Trichrome and phospho-histone H3 staining of pancreatic tumours from mice treated with vehicle (5.2% PEG-400/5.2% Tween80) or 0.2 mg $kg^{-1}$ CR-31 daily for 7 d. Tissues were harvested 2 h post final treatment. Scale bars, 50 μm.
j. Quantification of phospho-histone H3 positivity in tumours from mice treated with vehicle or CR-31 daily for 7 d. Tissues were harvested 2 h post final treatment. Data are mean +/– S.D., Student's *t*-test, n ≥ 7 fields of view. NS = not significant.

Source data are provided as a Source Data file.

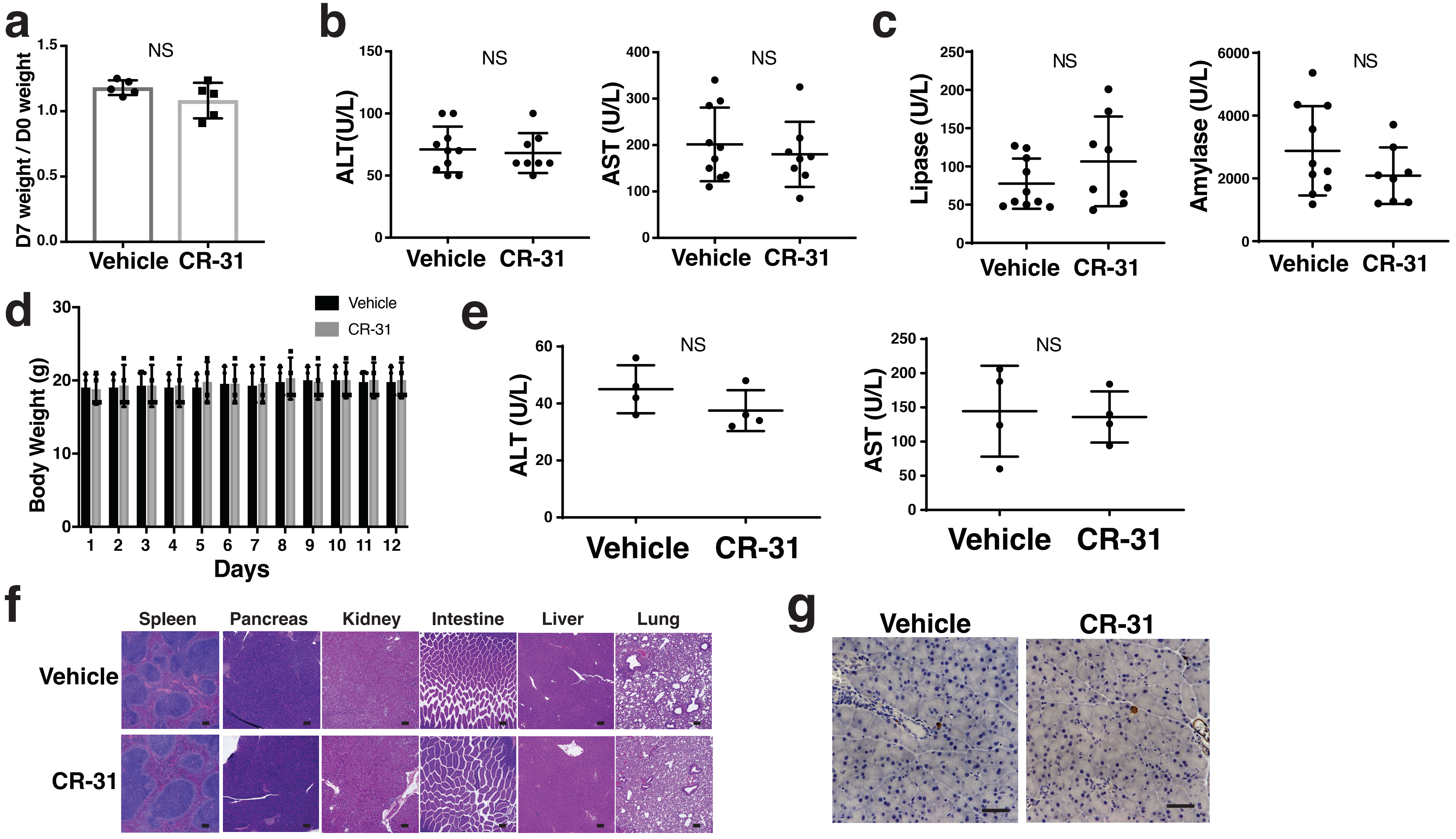


Supplementary Figure 2

**Supplementary Figure 2. CR-31 does not induce systemic toxicity in vivo**

a. Relative body weight of pancreatic tumour-bearing mice after 7 d of daily treatment with vehicle (n = 5) or 0.2 mg $kg^{-1}$ CR-31 (n = 5). Data are mean +/– S.D., Student's *t*-test. NS = not significant.
b. Circulating aspartate aminotransferase (AST) and alanine aminotransferase (ALT) levels in pancreatic tumour-bearing mice after 7 d of daily treatment with vehicle (n = 10) or 0.2 mg $kg^{-1}$ CR-31 (n = 8). Data are mean +/– S.D., Student's *t*-test. NS = not significant.
c. Circulating lipase and amylase levels in pancreatic tumour-bearing mice after 7 d of daily treatment with vehicle (n = 10) or 0.2 mg $kg^{-1}$ CR-31 (n = 8). Data are mean +/– S.D., Student's *t*-test. NS = not significant.
d. Daily body weight of C57Bl/6J mice upon 12 d of daily treatment with vehicle (n = 4) or 0.2 mg $kg^{-1}$ CR-31 (n = 4). 2 males and 2 females used per treatment arm. Data are mean +/– S.D.
e. Circulating aspartate aminotransferase (AST) and alanine aminotransferase (ALT) levels in C57Bl/6J mice after 12 d of daily treatment with vehicle (n = 4) or 0.2 mg $kg^{-1}$ CR-31 (n = 4). 2 males and 2 females used per treatment arm. Data are mean +/– S.D. Student's *t*-test. NS = not significant.
f. Hematoxylin and Eosin (H&E) staining of various organs from C57Bl/6J mice treated with vehicle or 0.2 mg $kg^{-1}$ CR-31 daily for 12 d. Tissues were harvested 2 h post final treatment. Scale bars, 100 µm.
g. Cleaved caspase 3 staining of the exocrine pancreas from C57Bl/6J mice treated with vehicle or 0.2 mg $kg^{-1}$ CR-31 daily for 12 d. Tissues were harvested 2 h post final treatment. Representative image of 4 mice per treatment group. Scale bars, 50 µm.

Source data are provided as a Source Data file.

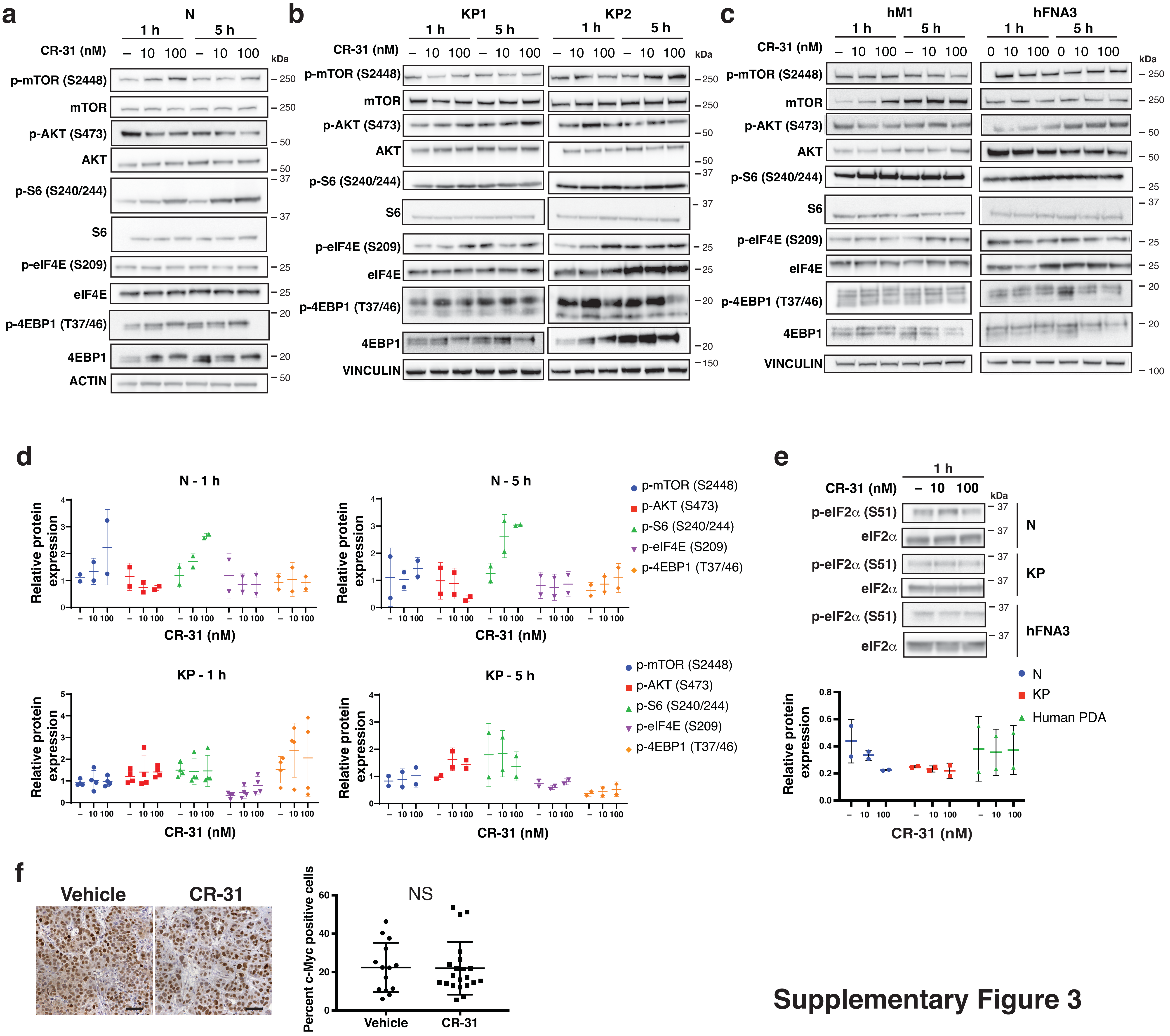


Supplementary Figure 3

**Supplementary Figure 3. Effect of CR-31 in growth factor signaling pathways**

a. Immunoblot analysis for AKT/mTOR and MNK1/2 signaling in N organoids upon treatment with the indicated concentrations of CR-31 for 1 and 5 h. ACTIN, loading control.
b. Immunoblot analysis for AKT/mTOR and MNK1/2 signaling in KP organoids upon treatment with the indicated concentrations of CR-31 for 1 and 5 h. VINCULIN, loading control.
c. Immunoblot analysis for AKT/mTOR and MNK1/2 signaling in patient-derived PDA cell lines upon treatment with the indicated concentrations of CR-31 for 1 and 5 h. VINCULIN, loading control.
d. Quantification of the indicated phospho-proteins normalized to the total level of each respective protein. Data are mean +/– S.D.
e. Immunoblot analysis of p-eIF2α in murine organoids (N and KP) and patient-derived PDA cell line (hFNA3) upon treatment with the indicated concentrations of CR-31 for 1 h (top). Total eIF2α, loading control. Quantification of p-eIF2α normalized to total eIF2α (bottom). Data are mean +/– S.D.
f. Immunohistochemistry of c-MYC (left) in representative tumours from mice treated daily for 7 d with vehicle or with 0.2 mg $kg^{-1}$ CR-31. Tissues were harvested 2 h post final treatment. Scale bars, 50 µm. Quantification of the percentage of c-MYC positive cells (right). Data are mean +/– S.D., Student's *t*-test, $n \geq 3$ fields of view. NS = not significant.

Source data are provided as a Source Data file.

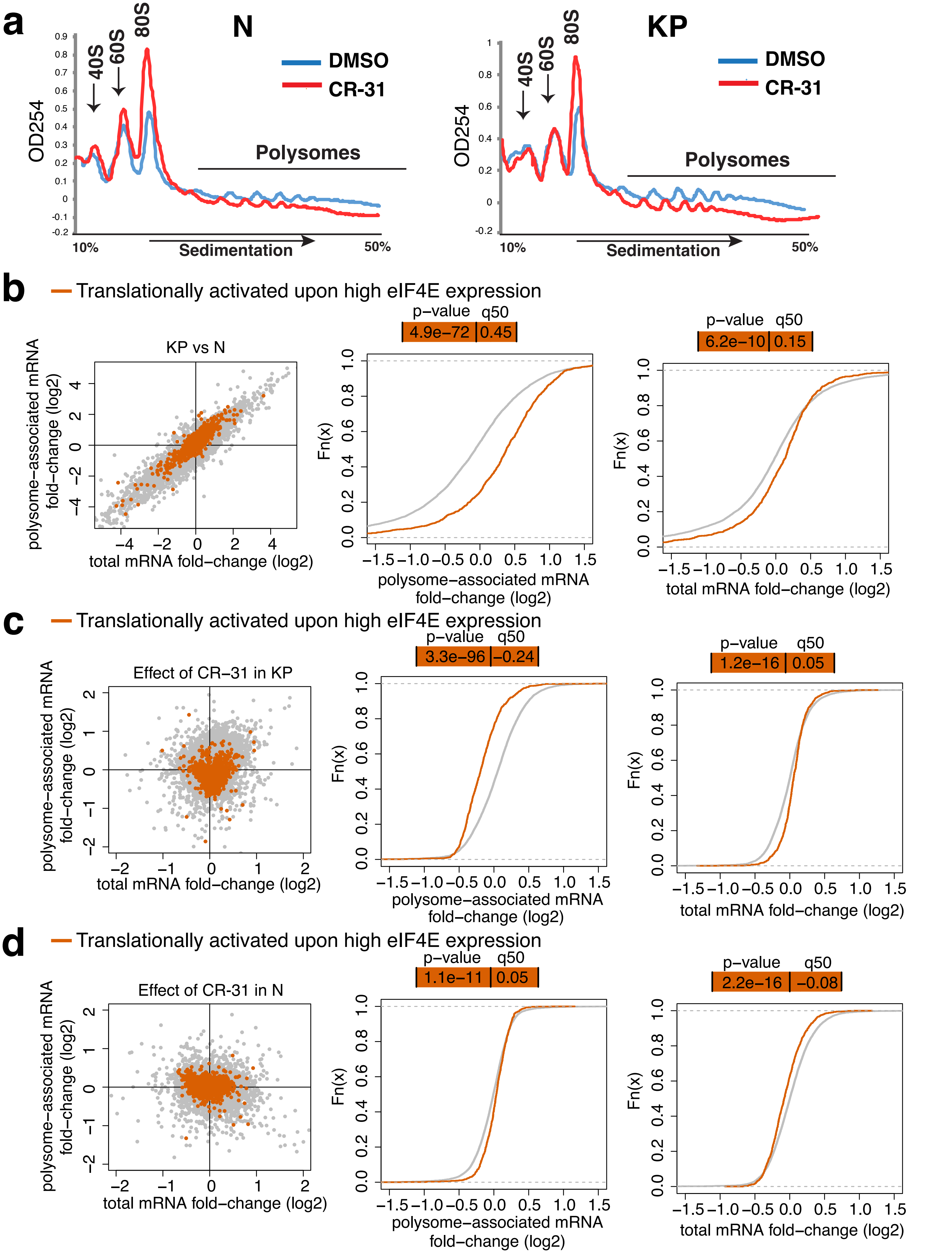


# Supplementary Figure 4

**Supplementary Figure 4. Polysome profile analysis of translation activity**

a. Polysome profiles of N and KP organoids treated with vehicle (DMSO) or 10 nM CR-31 for 1 h. Absorbance at 254 nm is shown as a function of sedimentation. Representative profiles from three biological replicates.
b. Scatterplot of log2 fold-changes for polysome-associated mRNA and total mRNA (left), an empirical cumulative distribution (ecdf) of log2 fold-changes for polysome-associated mRNA (middle) and total mRNA (right) for gene expression differences between untreated N and KP organoids. Transcripts whose translation was activated under high eIF4E expression [1] are represented in orange. Wilcoxon rank-sum test p-values comparing regulation of subsets to background are indicated together with the log2 fold-changes difference between these subsets to the background at the $50^{th}$ percentile (q50) of the ecdf.
c. Scatterplot of log2 fold-changes for polysome-associated mRNA and total mRNA (left), an empirical cumulative distribution (ecdf) of log2 fold-changes for polysome-associated mRNA (middle) and total mRNA (right) for gene expression differences between vehicle (DMSO) and CR-31 treated KP organoids. Transcripts whose translation was activated under high eIF4E expression [1] are represented in orange. Wilcoxon rank-sum test p-values comparing regulation of subsets to background are indicated together with the log2 fold-changes difference between these subsets to the background at the $50^{th}$ percentile (q50) of the ecdf.
d. Scatterplot of log2 fold-changes for polysome-associated mRNA and total mRNA (left), an empirical cumulative distribution (ecdf) of log2 fold-changes for polysome-associated mRNA (middle) and total mRNA (right) for gene expression differences between vehicle (DMSO) and CR-31 treated N organoids. Transcripts whose translation was activated under high eIF4E expression [1] are represented in orange. Wilcoxon rank-sum test p-values comparing regulation of subsets to background are indicated together with the log2 fold-changes difference between these subsets to the background at the $50^{th}$ percentile (q50) of the ecdf.

Source data are provided as a Source Data file.

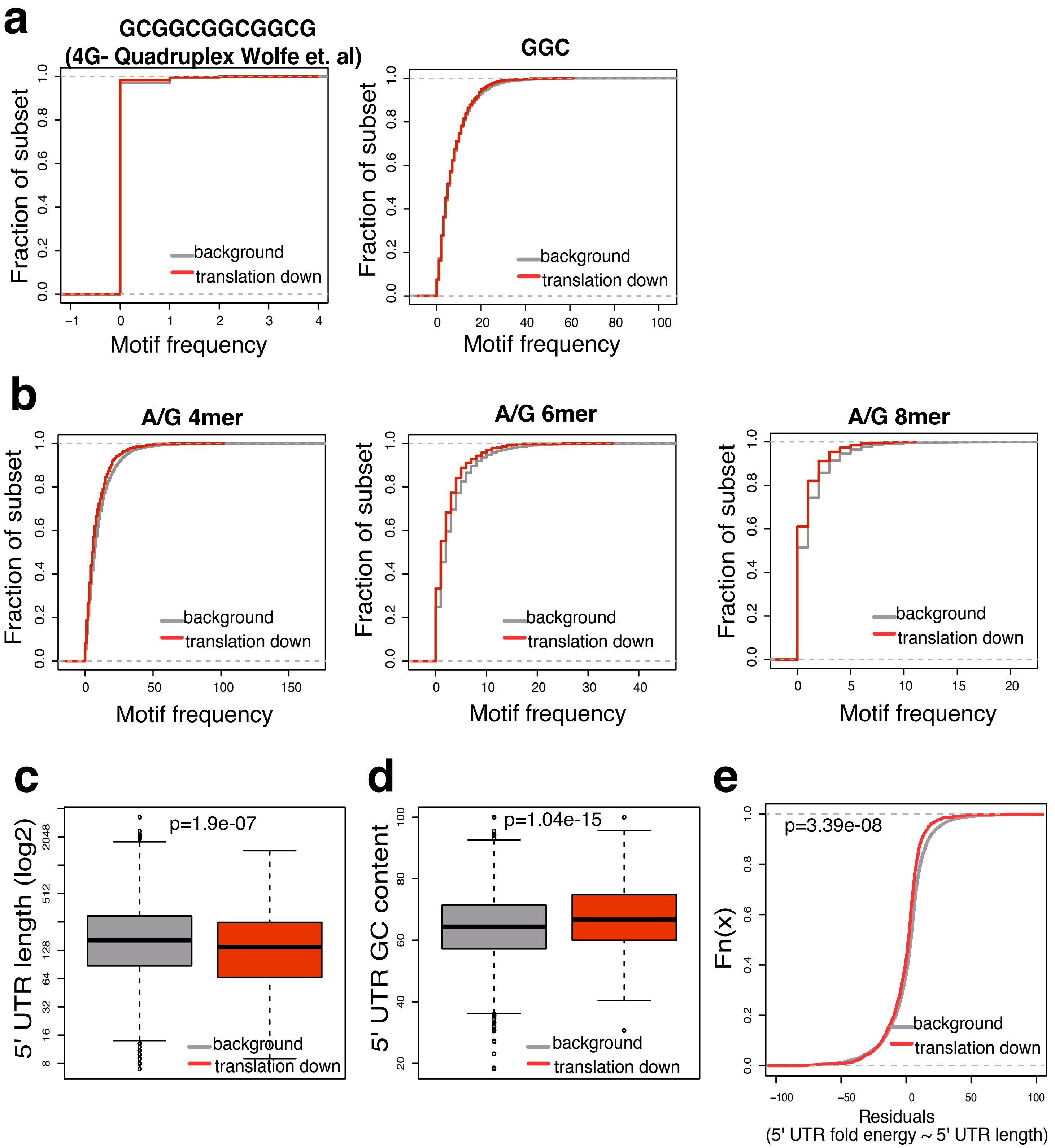


# Supplementary Figure 5

**Supplementary Figure 5. 5' UTR features of CR-31-sensitive transcripts**

a. Frequency of 4G-quadruplex motif identified by Wolfe et al. and GGC stretches in the 5' UTRs of translationally supressed mRNAs upon CR-31treatment in KP organoids (red) and unregulated transcripts (background, grey).
b. Frequency of polypurine stretches (A/G mers) with increasing lengths (left to right, 4-8) in the 5' UTRs of translationally supressed mRNAs upon CR31 treatment in KP organoids (red) and unregulated transcripts (background, grey).
c. Boxplot comparing 5' UTR lengths of sets of transcripts whose translation was suppressed (translation down) in KP organoids upon CR-31 treatment to unregulated transcripts (background, grey). Horizontal line indicates the median and the lower and upper box indicate the first and third quartiles. The whiskers extend from the box to the most extreme value but not further than +/- 1.5 * inter-quartile range (IQR) from the box. The notches extend to 1.58 * IQR/$\sqrt{n}$ where $n$ is the number of data points. Data exceeding the whiskers are plotted as points. Wilcoxon rank-sum test $p$-values for the indicated comparisons are shown.
d. Boxplot comparing 5' UTR GC content of sets of transcripts whose translation was suppressed (translation down) in KP organoids upon CR-31 treatment to unregulated transcripts (background, grey). Wilcoxon rank-sum test $p$-values for the indicated comparisons are shown.
e. An empirical cumulative distribution function (ecdf) of residuals from a linear model adjusting 5' UTR folding energy for 5' UTR length. Negative values indicate more stable 5' UTR than expected by the length whereas more positive values indicate a less stable 5' UTR than expected by the length. The ecdf for transcript translationally suppressed (translation down) upon CR-31 treatment in KP organoids are compared to background transcripts. Wilcoxon rank-sum test $p$-values for the indicated comparisons are shown. 5' UTR fold energies are in kcal $mol^{-1}$.

Source data are provided as a Source Data file.

KP organoids vs Normal organoids – Upregulated at the Polysome-associated mRNA level – gage analysis

Kegg Pathways

| Pathway name | p.geomean | stat.mean | p.val | q.val | set.size | exp1 |
|---|---|---|---|---|---|---|
| mmu00190 Oxidative phosphorylation | 9.82658E-20 | 0.479456605 | 9.82658E-20 | 2.83988E-17 | 96 | 9.82658E-20 |
| mmu05012 Parkinson's disease | 1.59229E-18 | 0.46636474 | 1.59229E-18 | 2.30086E-16 | 95 | 1.59229E-18 |
| mmu05010 Alzheimer's disease | 2.85916E-16 | 0.381965845 | 2.85916E-16 | 2.75432E-14 | 124 | 2.85916E-16 |
| mmu03010 Ribosome | 1.37869E-15 | 0.382752906 | 1.37869E-15 | 9.96104E-14 | 118 | 1.37869E-15 |
| mmu05016 Huntington's disease | 1.27753E-14 | 0.341307432 | 1.27753E-14 | 7.38415E-13 | 139 | 1.27753E-14 |
| mmu03030 DNA replication | 1.31359E-09 | 0.566069552 | 1.31359E-09 | 6.32715E-08 | 32 | 1.31359E-09 |
| mmu01200 Carbon metabolism | 4.42631E-07 | 0.283121736 | 4.42631E-07 | 1.82744E-05 | 92 | 4.42631E-07 |
| mmu04723 Retrograde endocannabinoid signaling | 1.02784E-06 | 0.300231601 | 1.02784E-06 | 3.54238E-05 | 77 | 1.02784E-06 |
| mmu04932 Non-alcoholic fatty liver disease (NAFLD) | 1.10316E-06 | 0.242341624 | 1.10316E-06 | 3.54238E-05 | 118 | 1.10316E-06 |
| mmu03460 Fanconi anemia pathway | 2.77867E-06 | 0.40568512 | 2.77867E-06 | 8.03034E-05 | 39 | 2.77867E-06 |
| mmu00983 Drug metabolism - other enzymes | 3.4711E-06 | 0.335846886 | 3.4711E-06 | 9.11952E-05 | 56 | 3.4711E-06 |
| mmu03013 RNA transport | 5.71954E-06 | 0.216708579 | 5.71954E-06 | 0.000137746 | 130 | 5.71954E-06 |
| mmu01230 Biosynthesis of amino acids | 1.20133E-05 | 0.327687582 | 1.20133E-05 | 0.000267065 | 53 | 1.20133E-05 |
| mmu05322 Systemic lupus erythematosus | 1.4878E-05 | 0.276808671 | 1.4878E-05 | 0.000305625 | 73 | 1.4878E-05 |
| mmu04110 Cell cycle | 1.58629E-05 | 0.22831971 | 1.58629E-05 | 0.000305625 | 107 | 1.58629E-05 |
| mmu00240 Pyrimidine metabolism | 2.07496E-05 | 0.367776999 | 2.07496E-05 | 0.000374789 | 40 | 2.07496E-05 |
| mmu03050 Proteasome | 2.81409E-05 | 0.367146045 | 2.81409E-05 | 0.000467898 | 39 | 2.81409E-05 |
| mmu00980 Metabolism of xenobiotics by cytochrome P450 | 2.91424E-05 | 0.35751261 | 2.91424E-05 | 0.000467898 | 41 | 2.91424E-05 |
| mmu00480 Glutathione metabolism | 3.31132E-05 | 0.312681 | 3.31132E-05 | 0.00050367 | 53 | 3.31132E-05 |
| mmu05034 Alcoholism | 4.28449E-05 | 0.210175747 | 4.28449E-05 | 0.000619109 | 115 | 4.28449E-05 |
| mmu04114 Oocyte meiosis | 7.08884E-05 | 0.243717193 | 7.08884E-05 | 0.000975559 | 81 | 7.08884E-05 |
| mmu00982 Drug metabolism - cytochrome P450 | 8.27001E-05 | 0.361899755 | 8.27001E-05 | 0.001086379 | 36 | 8.27001E-05 |
| mmu00270 Cysteine and methionine metabolism | 0.000130889 | 0.343567508 | 0.000130889 | 0.001644653 | 38 | 0.000130889 |
| mmu03440 Homologous recombination | 0.000143103 | 0.37836616 | 0.000143103 | 0.001723202 | 31 | 0.000143103 |
| mmu00650 Butanoate metabolism | 0.000150111 | 0.509244727 | 0.000150111 | 0.001735287 | 17 | 0.000150111 |
| mmu00970 Aminoacyl-tRNA biosynthesis | 0.000169561 | 0.330014025 | 0.000169561 | 0.001884736 | 40 | 0.000169561 |
| mmu05014 Amyotrophic lateral sclerosis (ALS) | 0.00020344 | 0.35939155 | 0.00020344 | 0.002177566 | 33 | 0.00020344 |
| mmu03040 Spliceosome | 0.000243019 | 0.187976718 | 0.000243019 | 0.002508305 | 119 | 0.000243019 |
| mmu00030 Pentose phosphate pathway | 0.000281132 | 0.413165266 | 0.000281132 | 0.002801621 | 24 | 0.000281132 |
| mmu04914 Progesterone-mediated oocyte maturation | 0.00031864 | 0.244040112 | 0.00031864 | 0.003069568 | 68 | 0.00031864 |
| mmu00860 Porphyrin and chlorophyll metabolism | 0.000344923 | 0.399740874 | 0.000344923 | 0.003215568 | 25 | 0.000344923 |
| mmu05204 Chemical carcinogenesis | 0.00039679 | 0.302348011 | 0.00039679 | 0.003583513 | 43 | 0.00039679 |
| mmu03420 Nucleotide excision repair | 0.00061633 | 0.32545343 | 0.00061633 | 0.00539756 | 35 | 0.00061633 |
| mmu00520 Amino sugar and nucleotide sugar metabolism | 0.000951803 | 0.311338941 | 0.000951803 | 0.007917308 | 36 | 0.000951803 |
| mmu00051 Fructose and mannose metabolism | 0.000958843 | 0.352714536 | 0.000958843 | 0.007917308 | 28 | 0.000958843 |
| mmu03430 Mismatch repair | 0.001107049 | 0.447745295 | 0.001107049 | 0.008740089 | 17 | 0.001107049 |
| mmu00620 Pyruvate metabolism | 0.001118973 | 0.336976703 | 0.001118973 | 0.008740089 | 30 | 0.001118973 |
| mmu03410 Base excision repair | 0.001708057 | 0.343927852 | 0.001708057 | 0.01299022 | 27 | 0.001708057 |
| mmu01524 Platinum drug resistance | 0.001880619 | 0.217054609 | 0.001880619 | 0.013935866 | 67 | 0.001880619 |
| mmu00230 Purine metabolism | 0.002221635 | 0.19370566 | 0.002221635 | 0.016051315 | 82 | 0.002221635 |
| mmu00260 Glycine, serine and threonine metabolism | 0.00248439 | 0.340005118 | 0.00248439 | 0.017511917 | 26 | 0.00248439 |
| mmu01210 2-Oxocarboxylic acid metabolism | 0.002768877 | 0.476203289 | 0.002768877 | 0.019052509 | 13 | 0.002768877 |
| mmu00010 Glycolysis / Gluconeogenesis | 0.00311417 | 0.253740244 | 0.00311417 | 0.020930119 | 45 | 0.00311417 |
| mmu01212 Fatty acid metabolism | 0.004356004 | 0.238464912 | 0.004356004 | 0.028611027 | 48 | 0.004356004 |
| mmu04146 Peroxisome | 0.006843995 | 0.197887386 | 0.006843995 | 0.043953659 | 64 | 0.006843995 |
| mmu00900 Terpenoid backbone biosynthesis | 0.008317736 | 0.35531036 | 0.008317736 | 0.052257082 | 19 | 0.008317736 |
| mmu00510 N-Glycan biosynthesis | 0.010520949 | 0.227934377 | 0.010520949 | 0.064692642 | 44 | 0.010520949 |
| mmu00630 Glyoxylate and dicarboxylate metabolism | 0.011951083 | 0.304009104 | 0.011951083 | 0.071955477 | 24 | 0.011951083 |
| mmu00040 Pentose and glucuronate interconversions | 0.012766734 | 0.339045181 | 0.012766734 | 0.075297679 | 19 | 0.012766734 |
| mmu00670 One carbon pool by folate | 0.014591161 | 0.363715885 | 0.014591161 | 0.084336911 | 16 | 0.014591161 |
| mmu00280 Valine, leucine and isoleucine degradation | 0.016055764 | 0.222188823 | 0.016055764 | 0.090982665 | 42 | 0.016055764 |

CR-31-treated KP organoids - Down regulated at the Polysome-associated mRNA level - gage analysis

Kegg Pathways

| Pathway name | p.geomean | stat.mean | p.val | q.val | set.size | exp1 |
|---|---|---|---|---|---|---|
| mmu00190 Oxidative phosphorylation | 5.4359E-15 | 0.415351862 | 5.4359E-15 | 1.57098E-12 | 96 | 5.4359E-15 |
| mmu05012 Parkinson's disease | 2.61088E-10 | 0.342211824 | 2.61088E-10 | 3.77272E-08 | 95 | 2.61088E-10 |
| mmu03010 Ribosome | 1.60091E-09 | 0.294464991 | 1.60091E-09 | 1.45312E-07 | 118 | 1.60091E-09 |
| mmu05322 Systemic lupus erythematosus | 2.25103E-09 | 0.370482997 | 2.25103E-09 | 1.45312E-07 | 73 | 2.25103E-09 |
| mmu05034 Alcoholism | 2.51406E-09 | 0.29489943 | 2.51406E-09 | 1.45312E-07 | 115 | 2.51406E-09 |
| mmu05010 Alzheimer's disease | 4.07566E-08 | 0.263366413 | 4.07566E-08 | 1.96311E-06 | 124 | 4.07566E-08 |
| mmu04932 Non-alcoholic fatty liver disease (NAFLD) | 1.42852E-06 | 0.240046744 | 1.42852E-06 | 5.89774E-05 | 118 | 1.42852E-06 |
| mmu05016 Huntington's disease | 1.71724E-06 | 0.219856598 | 1.71724E-06 | 6.20354E-05 | 139 | 1.71724E-06 |
| mmu04723 Retrograde endocannabinoid signaling | 1.85009E-05 | 0.266914049 | 1.85009E-05 | 0.000594086 | 77 | 1.85009E-05 |
| mmu00480 Glutathione metabolism | 0.000395019 | 0.272532089 | 0.000395019 | 0.011416048 | 53 | 0.000395019 |
| mmu04260 Cardiac muscle contraction | 0.001891527 | 0.264472505 | 0.001891527 | 0.049695578 | 45 | 0.001891527 |
| mmu00240 Pyrimidine metabolism | 0.002186797 | 0.27719106 | 0.002186797 | 0.052665369 | 40 | 0.002186797 |
| mmu00980 Metabolism of xenobiotics by cytochrome P450 | 0.003770062 | 0.261345418 | 0.003770062 | 0.083811378 | 41 | 0.003770062 |

Supplementary Figure 6

**Supplementary Figure 6. Pathway analysis of CR-31-sensitive transcripts**

Pathway enrichment analysis for mRNAs exhibiting increased polysome association in KP relative to N organoids, and those downregulated upon CR-31 treatment in KP organoids.

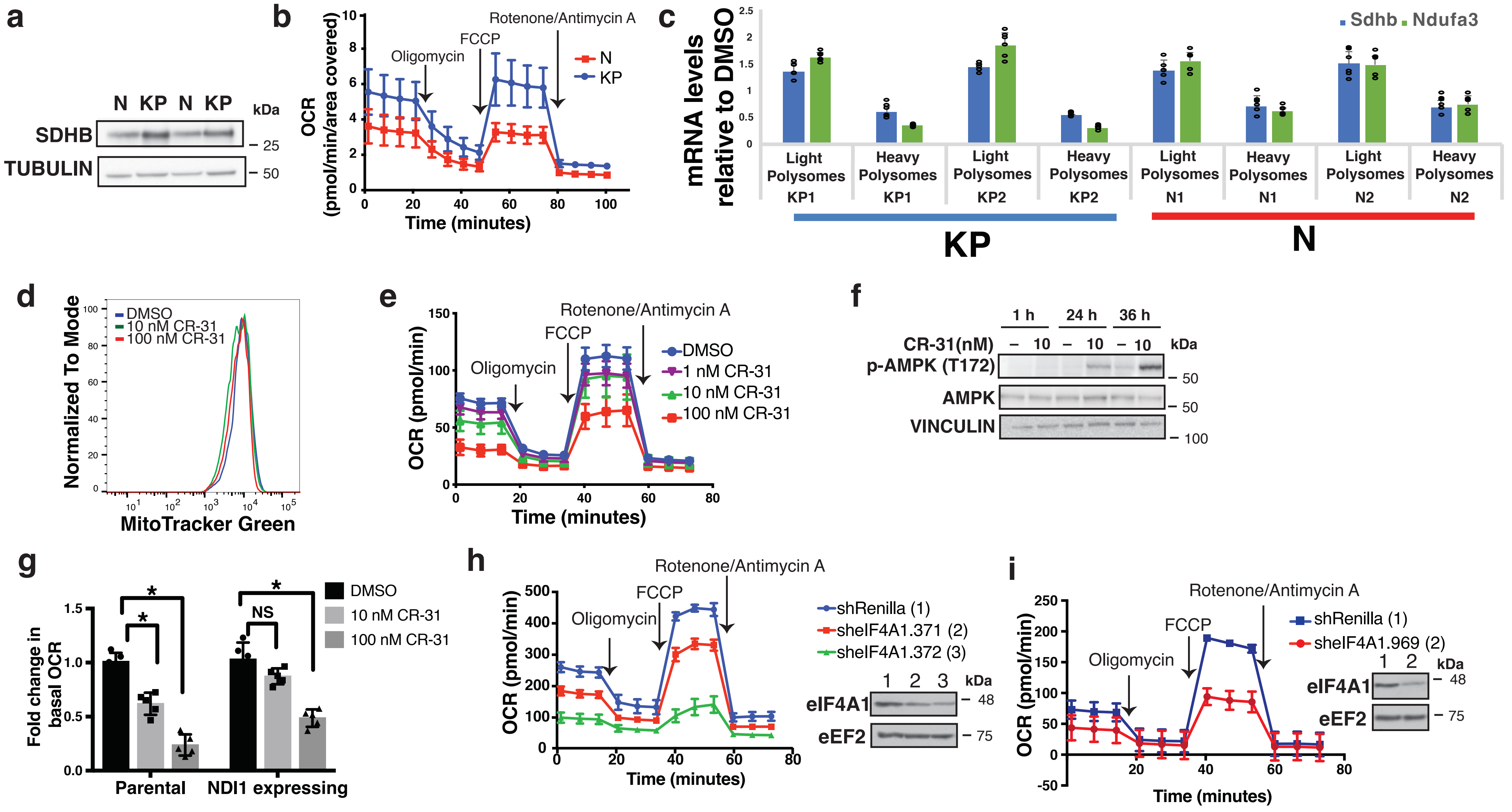


**Supplementary Figure 7**

**Supplementary Figure 7. CR-31 inhibits OxPHOS in PDA**

a. SDHB levels in N and KP organoids. TUBULIN, loading control.
b. OCR levels in N and KP organoids following the addition of oligomycin, FCCP and rotenone/antimycin A (0.5 μM). Data normalized to covered area measured using CellInSight CX5. Data are mean +/– S.D., n = 5.
c. Amounts of mRNA (quantified by RT-qPCR) of transcripts encoding electron transport chain proteins in light (<3 ribosomes) or heavy (≥3 ribosomes) polysome fractions from N or KP organoids treated with 10 nM CR-31 for 1 h relative to vehicle (DMSO). Data are mean +/– S.D., n=6 Data from two biological replicates are shown.
d. MitoTracker green labelling of patient-derived PDA cells upon treatment with vehicle (DMSO) or the indicated concentrations of CR-31 for 24 h. Representative from 3 biological replicates.
e. OCR of patient-derived PDA cells treated with vehicle (DMSO) or the indicated concentrations of CR-31 for 5 h under basal conditions or following the addition of oligomycin, FCCP, rotenone/antimycin A. Data are mean +/– S.D., n = 6.
f. Immunoblot analysis of AMPK activation status in KP organoids upon vehicle (DMSO) or 10 nM CR-31 treatment. VINCULIN, loading control.
g. Rates of basal oxidative phosphorylation reflected by OCR was measured in primary murine pancreatic cancer cell lines with and without NDI1 expression. Data are mean +/– S.D., n = 5. *, $p < 0.05$ compared to DMSO treatment. NS = not significant.
h. OCR of primary murine pancreatic cancer cell lines expressing sh*Renilla* or two hairpins against *eIF4A1* (sheIF4A1.371, sheIF4A1.372) on day 3 after infection under basal conditions or following the addition of oligomycin, FCCP, or Rotenone/Antimycin A. Data are mean +/– S.D., n = 5. Inset, immunoblot of eIF4A1 to confirm knockdown. eEF2, loading control.
i. OCR of patient-derived PDA cell lines expressing sh*Renilla* or hairpin against *eIF4A1* (shEIF4A1.969) on day 3 after infection, under basal conditions or following the addition of oligomycin, FCCP, and rotenone/antimycin A. Data are mean +/– S.D., n = 5. Inset, immunoblot of eIF4A1 to confirm knockdown. eEF2, loading control.

Source data are provided as a Source Data file.

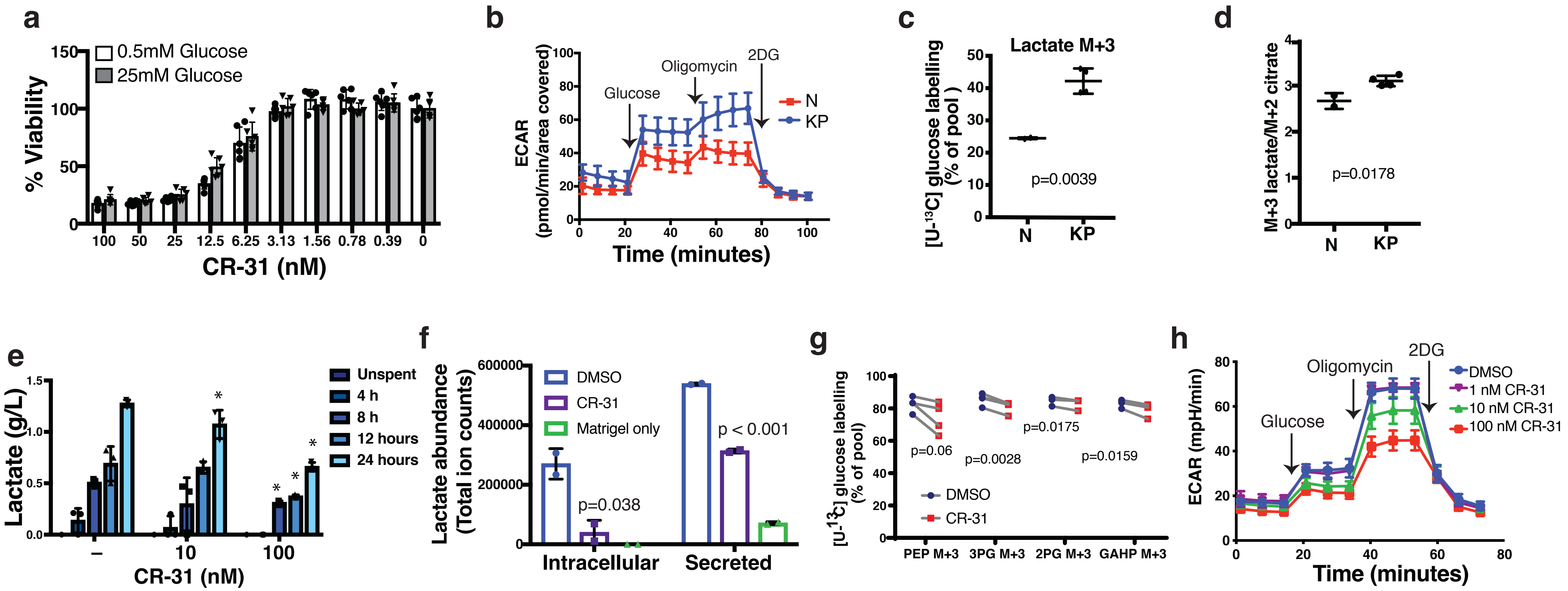


**Supplementary Figure 8**

**Supplementary Figure 8. CR-31 inhibits glucose metabolism in PDA**

a. Cell viability of patient-derived PDA cell lines after 72 h of treatment with increasing concentrations of CR-31 when cultured in 25 mM or 0.5 mM of glucose. Data are mean +/– S.D., n = 6.
b. ECAR of N and KP organoids at steady state following the addition of glucose (10 mM), Oligomycin (1 μM) and the glucose analog, 2-deoxyglucose, 2DG (50 mM). Data normalized to covered area. Data are mean +/– S.D., n = 5.
c. $^{13}C_6$-glucose tracing of N (n = 2) and KP organoids (n = 4) to determine the contribution of glucose to glycolysis. M+3 labelled lactate is the end product of $^{13}C_6$-glucose in the glycolytic pathway. Data are mean +/– S.D., Student's *t* test.
d. Ratio of lactate (M+3) to citrate (M+2) for N (n = 2) and KP organoids (n = 4) to determine the contribution of glucose to glycolysis relative to the TCA cycle. Data are mean +/– S.D., Student's *t* test.
e. Lactate concentration in media spent by KP organoids upon treatment with vehicle (DMSO) or CR-31. Data are mean +/– S.D., Student's *t*-test, n = 3. *, $p < 0.05$ CR-31 compared to DMSO treatment at each timepoint.
f. Steady state lactate levels of 2 biological replicates of KP organoids treated with vehicle (DMSO) or 100 nM CR-31 for 5 h. Data are mean +/– S.D., Student's *t*-test, CR-31 compared to DMSO treatment.
g. $^{13}C_6$-glucose tracing of 4 patient-derived PDA cell lines treated with vehicle (DMSO) or 100 nM CR-31 for 5 h to determine the contribution of glucose to all detected glycolytic intermediates: M+3 labelling on phosphoenolpyruvate (PEP), 3-phospho-D-glycerate (3PG), 2-phosphoglyceric acid (2PG), and glyceraldehye 3-phosphate (GAHP). Data are mean +/– S.D., Student's paired *t*-test.
h. ECAR of in patient-derived PDA cell lines after 5 h of treatment with vehicle (DMSO) or the indicated concentrations of CR-31. ECAR was measured before and following the addition of glucose (10 mM), oligomycin (1 μM) and the glucose analog, 2-deoxyglucose, 2DG (50 mM). Data are mean +/– S.D., n = 6.

Source data are provided as a Source Data file.

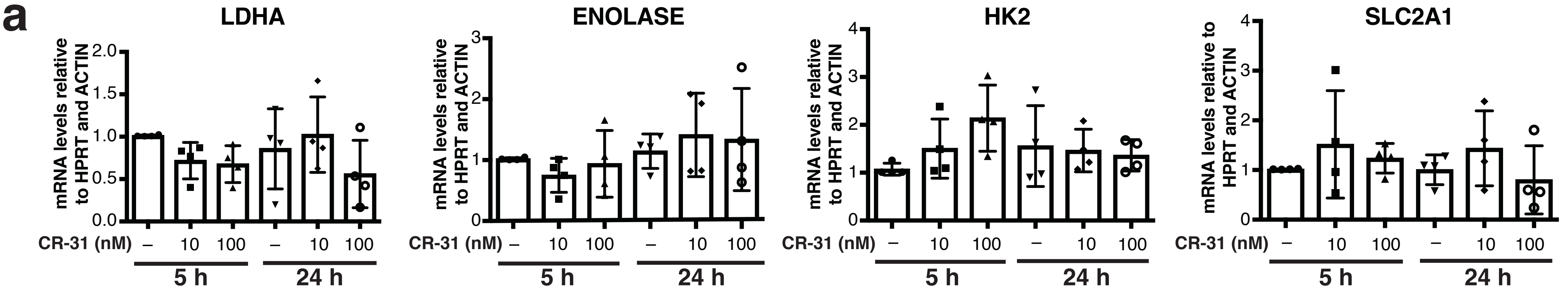


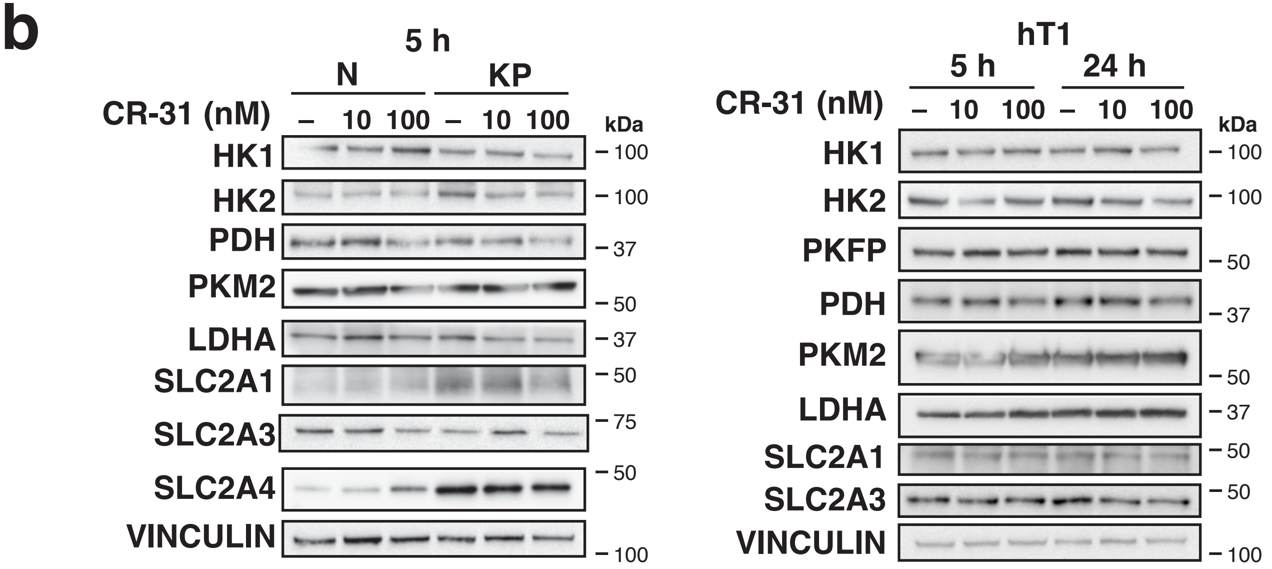


Supplementary Figure 9

**Supplementary Figure 9. Effect of CR-31 on the expression of glycolytic enzymes**

a. RT-qPCR analysis of mRNAs encoding glycolytic enzymes in 4 patient-derived PDA cell lines upon treatment with the indicated concentration and duration of CR-31. Data are mean +/– S.D., n = 3. Each data point plotted is average from every biological replicate.
b. Immunoblot analysis of glycolytic enzymes in murine organoids (left) and patient-derived PDA cell lines (right) upon treatment with the indicated concentration of CR-31. VINCULIN, loading control.

Source data are provided as a Source Data file.

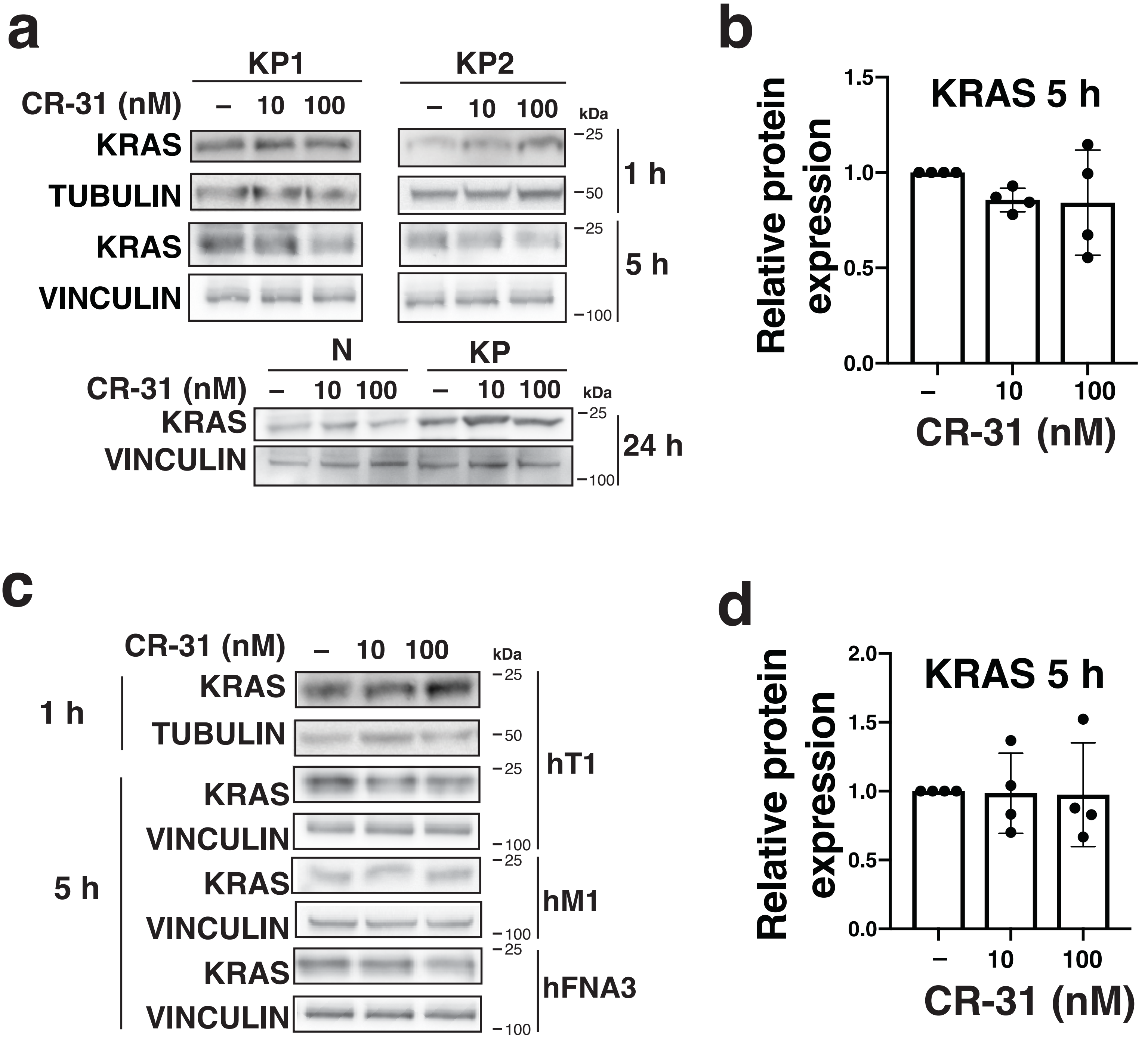


Supplementary Figure 10

**Supplementary Figure 10. Effect of CR-31 on the expression of KRAS**

a. Immunoblot analysis of KRAS expression in murine organoids upon vehicle (DMSO) or CR-31 treatment for the indicated amount of time. TUBULIN and VINCULIN, loading controls.
b. Quantification of KRAS in murine organoids normalized to loading control. Data are mean +/– S.D., Student's *t* test. n = 4 biological replicates.
c. Immunoblot analysis and quantification of KRAS expression in patient-derived PDA cell lines (right) upon DMSO or CR-31 treatment for the indicated amount of time. TUBULIN and VINCULIN, loading controls.
d. Quantification of KRAS in patient-derived PDA cell lines normalized to loading control. Data are mean +/– S.D., Student's *t* test. n = 4 biological replicates.

Source data are provided as a Source Data file.

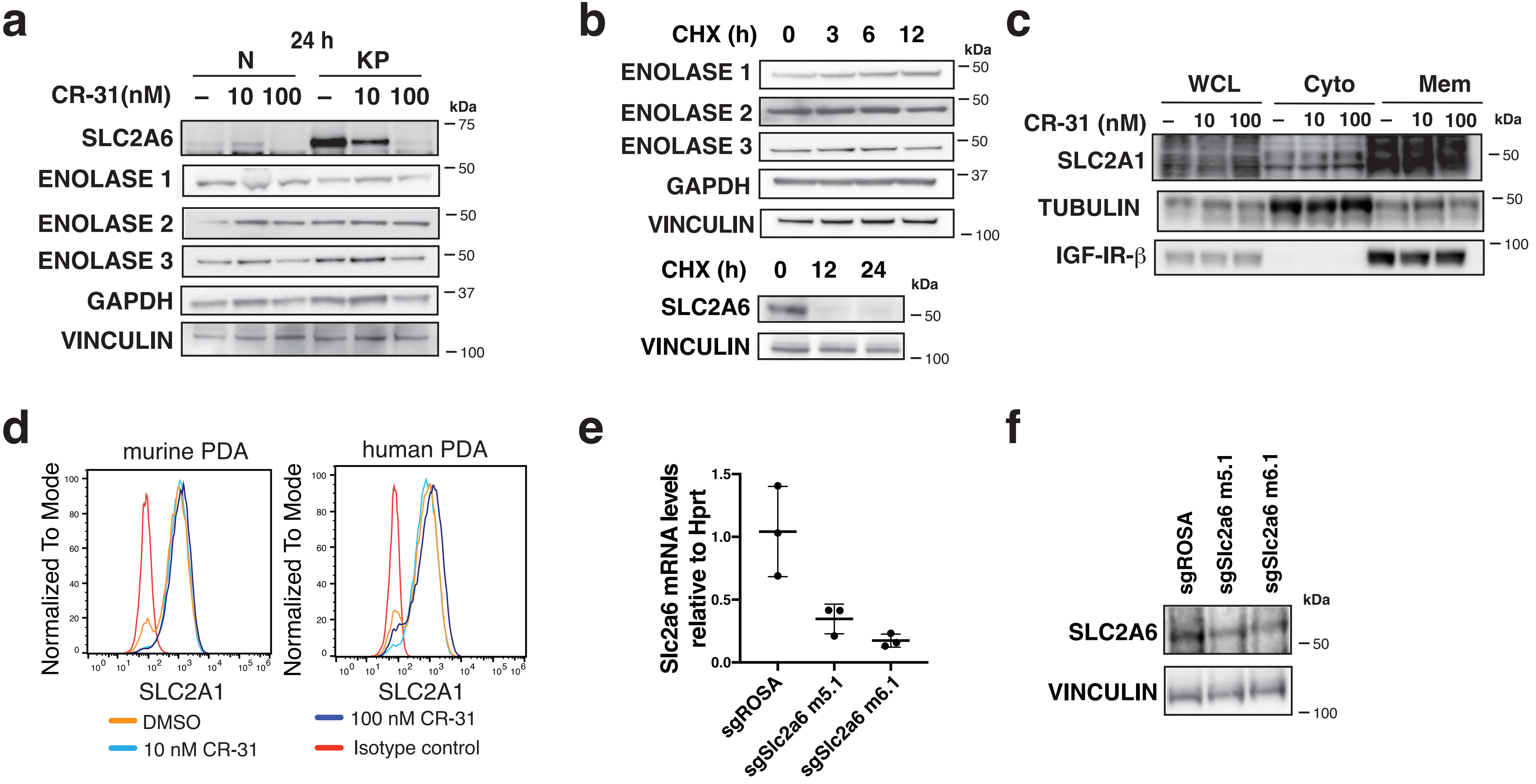


**Supplementary Figure 11**

**Supplementary Figure 11. CR-31 translationally regulates SLC2A6**

a. Immunoblot analysis of SLC2A6, ENOLASE 1, ENOLASE 2, ENOLASE 3, and GAPDH in N and KP organoids upon vehicle (DMSO) or CR-31 treatment for 24 h. VINCULIN, loading control.
b. Patient-derived PDA cell lines were exposed to 100 μg $ml^{-1}$ cycloheximide for the indicated amount of time. VINCULIN, loading control.
c. Immunoblot of SLC2A1 expression in whole cell lysates (WCL), cytoplasmic (Cyto), and membrane (Mem) fractions of primary murine PDA cell lines. IGF-IR-β, membrane fraction loading control. TUBULIN, cytoplasmic loading control.
d. SLC2A1 membrane expression upon 5 h of CR-31 treatment quantified by flow cytometry.
e. RT-qPCR analysis of *Slc2a6* mRNA expression in Cas9-expressing primary murine pancreatic cancer cell lines with sgRNAs targeting the ROSA locus (sg*ROSA*) or different regions of the *Slc2a6* gene (sgSlc2a6 m5.1 and sgSlc2a6 m6.1). Data are mean +/– S.D., n = 3.
f. Immunoblot analysis of SLC2A6 upon expression of sgRNA against the *ROSA* locus or against *Slc2a6* in Cas9-expressing primary murine pancreatic cancer cell lines. Vinculin, loading control.

Source data are provided as a Source Data file.

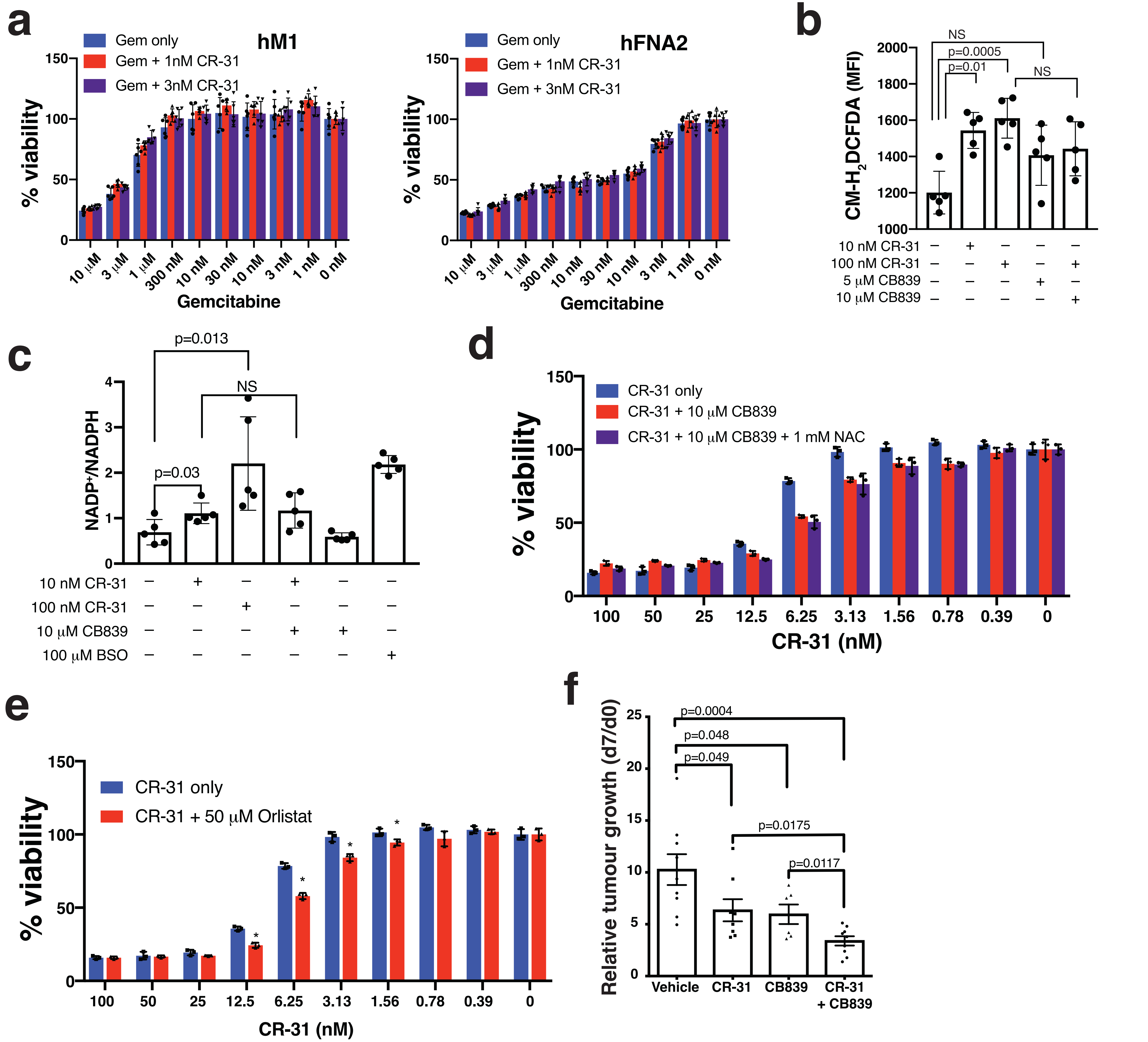


Supplementary Figure 12

**Supplementary Figure 12. Combined inhibition of eIF4A and metabolic pathways**

a. Cell viability of patient-derived pancreatic cancer cell lines upon a 72 h treatment with increasing concentrations of Gemcitabine and the indicated concentration of CR-31. Dotted lines, 95% confidence intervals, n = 6.
b. ROS levels in patient-derived PDA cell lines measured with CM-$H_2$DCFDA upon treatment with vehicle (DMSO) or the indicated concentrations of CR-31 and CB839 for 24 h. Data are mean +/– S.D., Student's *t* test, n = 5. NS = not significant.
c. Ratio of $NADP^+$/NADPH in patient-derived PDA cell lines upon treatment with vehicle (DMSO), CR-31, CB839, or L-buthionine sulfoximine (BSO) for 24 h. Data are mean +/– S.D., Student's *t* test, n = 5. NS = not significant.
d. Cell viability of primary murine pancreatic cancer cell upon treatment with increasing concentrations of CR-31 in the presence or absence of 10 μM CB839 and 1 mM of the ROS scavenger N-acetylcysteine (NAC) for 72 h. Data are mean +/– S.D., n = 3.
e. Cell viability of patient-derived PDA cell lines upon a 72 h treatment with increasing concentrations of CR-31 and the indicated concentration of Orlistat. Data are mean +/– S.D., Student's *t* test, n = 3. *, $p < 0.05$ compared to CR-31 treatment alone at each concentration.
f. Relative tumour volume of PDA-bearing mice (orthotopic transplants) after 7 d of treatment with vehicle (5.2% PEG-400/5.2% Tween80), CR-31 (0.2 mg $kg^{-1}$), CB-839 (200 mg $kg^{-1}$) or in combination. Relative tumour growth was estimated by comparison of tumour volume on 7 d post treatment to that at pre-enrollment (day 0) by ultrasound imaging. Student's *t*-test. (n = 9, vehicle; n = 8, CR-31; n = 6, CB839; n = 9, CR-31 + CB839).

Source data are provided as a Source Data file.

## REFERENCES

1 Svitkin, Y. V. *et al*. Eukaryotic translation initiation factor 4E availability controls the switch between cap-dependent and internal ribosomal entry site-mediated translation. *Mol Cell Biol* **25**, 10556-10565, (2005).
