# Supplementary material for: eIF4A supports an oncogenic translation program in pancreatic ductal adenocarcinoma
Source: Nat Commun. 2019 Nov 13;10:5151. doi: 10.1038/s41467-019-13086-5 (PMC6853918; doi:10.1038/s41467-019-13086-5)
Supplement: Supplementary file 7 — Reporting Summary [file 41467_2019_13086_MOESM7_ESM.pdf]

nature research

Corresponding author(s): *Double-blind peer review submissions: write DBPR and your manuscript number here instead of author names.*

Last updated by author(s): Jul 22, 2019

Reporting Summary

Nature Research wishes to improve the reproducibility of the work that we publish. This form provides structure for consistency and transparency in reporting. For further information on Nature Research policies, see Authors & Referees and the Editorial Policy Checklist.

Statistics

For all statistical analyses, confirm that the following items are present in the figure legend, table legend, main text, or Methods section.

n/a	Confirmed	
☐	☒	The exact sample size (n) for each experimental group/condition, given as a discrete number and unit of measurement
☐	☒	A statement on whether measurements were taken from distinct samples or whether the same sample was measured repeatedly
☐	☒	The statistical test(s) used AND whether they are one- or two-sided<br>*Only common tests should be described solely by name; describe more complex techniques in the Methods section.*
☒	☐	A description of all covariates tested
☒	☐	A description of any assumptions or corrections, such as tests of normality and adjustment for multiple comparisons
☐	☒	A full description of the statistical parameters including central tendency (e.g. means) or other basic estimates (e.g. regression coefficient) AND variation (e.g. standard deviation) or associated estimates of uncertainty (e.g. confidence intervals)
☐	☒	For null hypothesis testing, the test statistic (e.g. F, t, r) with confidence intervals, effect sizes, degrees of freedom and P value noted<br>*Give P values as exact values whenever suitable.*
☒	☐	For Bayesian analysis, information on the choice of priors and Markov chain Monte Carlo settings
☒	☐	For hierarchical and complex designs, identification of the appropriate level for tests and full reporting of outcomes
☒	☐	Estimates of effect sizes (e.g. Cohen's d, Pearson's r), indicating how they were calculated

Our web collection on statistics for biologists contains articles on many of the points above.

Software and code

Policy information about availability of computer code

Data collection

Provide a description of all commercial, open source and custom code used to collect the data in this study, specifying the version used OR state that no software was used.

Data analysis

Provide a description of all commercial, open source and custom code used to analyse the data in this study, specifying the version used OR state that no software was used.

For manuscripts utilizing custom algorithms or software that are central to the research but not yet described in published literature, software must be made available to editors/reviewers. We strongly encourage code deposition in a community repository (e.g. GitHub). See the Nature Research guidelines for submitting code & software for further information.

Data

Policy information about availability of data

All manuscripts must include a data availability statement. This statement should provide the following information, where applicable:

- Accession codes, unique identifiers, or web links for publicly available datasets
- A list of figures that have associated raw data
- A description of any restrictions on data availability

The datasets generated during and/or analysed during the current study are available in the GEO repository under the accession number GSE125380. Data can be previewed using the following access token: opozcaiczbizdul.

Field-specific reporting

Please select the one below that is the best fit for your research. If you are not sure, read the appropriate sections before making your selection.

☒ Life sciences ☐ Behavioural & social sciences ☐ Ecological, evolutionary & environmental sciences

For a reference copy of the document with all sections, see nature.com/documents/nr-reporting-summary-flat.pdf

Life sciences study design

All studies must disclose on these points even when the disclosure is negative.

Sample size	No sample size calculations were performed. Sample size was determined to be adequate based on the magnitude and consistency of measurable differences between groupd
Data exclusions	On principle, data were only excluded for failed experiments, reasons for which included microbial contamination
Replication	Replicate experiments were successful
Randomization	Pancreatic tumor-bearing mice were randomized into treatment arms for all in vivo therapeutic studies.
Blinding	Investigators were blinded to treatment groups during the analysis of all in vivo data. This included tumor volume measurements as well as the immunohistological staining and scoring of phospho-histone H3, c-Myc, cleaved caspase 3, amylase, lipase, AST, and ALT.

Reporting for specific materials, systems and methods

We require information from authors about some types of materials, experimental systems and methods used in many studies. Here, indicate whether each material, system or method listed is relevant to your study. If you are not sure if a list item applies to your research, read the appropriate section before selecting a response.

Materials & experimental systems

n/a	Involved in the study	
☐	☒	Antibodies
☐	☒	Eukaryotic cell lines
☒	☐	Palaeontology
☐	☒	Animals and other organisms
☒	☐	Human research participants
☒	☐	Clinical data

Methods

n/a	Involved in the study	
☐	☐	ChIP-seq
☐	☒	Flow cytometry
☐	☐	MRI-based neuroimaging

Antibodies

Antibodies used

4EBP1 (Cell Signaling Technology Cat# 9452, RRID:AB_331692) Lot # 10,
AMPK (Cell Signaling Technology Cat# 2793, RRID:AB_915794) (F6) Lot # 7,
Anti-Puromycin clone 12D10 (Millipore Cat# MABE343, RRID:AB_2566826) Lot # 3205917,
Cleaved Caspase 3 (Cell Signaling Technology Cat# 9664, RRID:AB_2070042) (D175) Lot # 21,
cMYC (Abcam Cat# ab32072, RRID:AB_731658) (Y69) Lot # GR295111-31,
EEF2 (Abcam Cat# ab75748, RRID:AB_1310165) (EP880Y) Lot # GR258629-5,
EIF2a (Cell Signaling Technology Cat# 5324, RRID:AB_10692650) (D7D3) Lot # 5,
EIF4A1 (Abcam #31217, RRID:AB_732122) Lot # GR3248712-1,
eIF4E (Cell Signaling Technology Cat# 2067, RRID:AB_2097675) (C46H6) Lot # 6 ,
Enolase 1 (Cell Signaling Technology Cat# 3810, RRID:AB_2246524) Lot # 2 ,
Enolase 2 (Cell Signaling Technology Cat# 9536, RRID:AB_2099308) Lot # 1
Enolase 3 (Abcam Cat# ab96334, RRID:AB_10680754) Lot #GR1786-9
GAPDH (Cell Signaling Technology Cat# 2118, RRID:AB_561053) (14C10) Lot # 10,
Glutaminase (Abcam Cat# ab93434, RRID:AB_10561964) Lot # GR3196690-2,
Glutaminase (ProteinTech Cat#12855-1-AP, RRID:AB_2110381) Lot # 0041545,
Hexokinase I (Cell Signaling Technology Cat# 2024, RRID:AB_2116996) (C35C4) Lot # 3,
Hexokinase II (Cell Signaling Technology Cat# 2867, RRID:AB_2232946) (C64G5) Lot # 5,
IGF-IR-B (Cell Signalling Cat# 9750, RRID:AB_10950969) (D23H3) Lot # 5
Kras (Abcam Cat# ab180772) Lot # GR3226768-1
LDHA (Cell Signaling Technology Cat# 3582, RRID:AB_2066887) (C4B5) Lot # 9,
mTOR (Cell Signaling Technology Cat# 2983, RRID:AB_2105622) (7C10) Lot # 12,
Nrf2 (homemade for mouse) affinity-purified rabbit-anti-full-length mouse Nrf2 antibody (made by JR Prigge, Montana State University) was a provided by Dr. Ed. Schmidt (Montana State University).
p4EBP1 (Cell Signaling Technology Cat# 3929, RRID:AB_10695878) (236B4) Lot # 20,

pAKT (Cell Signaling Technology Cat# 4060, RRID:AB_2315049) (D9E) Lot # 23,
pAMPK (Cell Signaling Technology Cat# 2535, RRID:AB_331250) (40H9) Lot # 21,
pan-Actin (Cell Signaling Technology Cat# 8456, RRID:AB_) (D18C11) Lot # 5,
pan-AKT (Cell Signaling Technology Cat# 4691, RRID:AB_) (C67E7) Lot # 17,
pEIF2a (Cell Signaling Technology Cat# 3398, RRID:AB_2096481) (D9G8) Lot # 6,
pEIF4E (Cell Signaling Technology Cat# 9741, RRID:AB_331677) Lot # 4,
PFKP (Cell Signaling Technology Cat# 8164, RRID:AB_2713957) (D4B2) Lot # 3,
phospho-Histone H3 (Cell Signaling Technology Cat# 9701, RRID:AB_331535) Lot # 17,
PKM2 (Cell Signaling Technology Cat# 4053, RRID:AB_1904096) (D78A4) Lot # 5,
pmTOR (Cell Signaling Technology Cat# 5536, RRID:AB_10691552) (D9C2) Lot # 5,
pS6 Ribosomal Protein (Cell Signaling Technology Cat# 5364, RRID:AB_10694233) (D68F8) Lot # 8,
Pyruvate Dehydrogenase (Cell Signaling Technology Cat# 3205, RRID:AB_2162926) (C54G1) Lot # 5,
S6 Ribosomal Protein (Cell Signaling Technology Cat# 2317, RRID:AB_2238583) (54D2) Lot # 4,
SDHB (Abcam Cat # ab14714, RRID:AB_301432) (SPM498) Lot # H0218,
Slc2a1 (Abcam Cat# 40084, RRID: AB_2190927) Lot # GR3213392-3,
Slc2a1 (Cell Signaling Technology Cat# 12939, RRID:AB_2687899) (D3J3A) Lot # 3,
Slc2a3 (Santa Cruz Biotechnology Cat# sc-74399, RRID:AB_1124975) (G5) Lot # K3018,
Slc2a4 (Cell Signaling Technology Cat# 2213, RRID: AB_823508) Lot # 7
Slc2a6 (Sigma-Aldrich Cat# HPA042272, RRID:AB_2677924) Lot # R39419,
Tubulin (Cell Signaling Technology Cat# 2148, RRID:AB_2288042) Lot # 7 ,
Vinculin (Cell Signaling Technology Cat# 4650, RRID:AB_10559207) Lot # 4.

Validation

The antibodies to be used in these studies will be well-characterized, validated commercial reagents from reliable sources (e.g., Abcam, Cell signaling) or generated through collaborators and reported in the scientific literature: Antibody raised against Nrf2: Polyclonal Nrf2 antibody was produced by immunizing rabbits with full-length recombinant mouse Nrf2 protein followed by affinity purification as described previously (Suvorova ES, Lucas O, Weisend CM, Rollins MF, Merrill GF, Capecchi MR, Schmidt EE. Cytoprotective Nrf2 pathway is induced in chronically txnrd 1-deficient hepatocytes. PloS one. 2009;4(7):e6158. doi: 10.1371/journal.pone.0006158. PubMed PMID: 19584930; PMCID: 2703566.) These antibodies were independently validated by the PI, demonstrating immunoreactivity to a 72 kDa protein in wildtype cells but not Nrf2 knockout cells).

Eukaryotic cell lines

Policy information about cell lines

Cell line source(s)

293T and PhoenixE cells were obtained from ATCC. Murine pancreatic ductal organoid cultures were generated and cultured as described in these two articles PMIDs: 25557080, 27477511. Patient-derived pancreatic cancer cells are gifts from Dr David Tuveson (CSHL). Pancreatic cancer associated fibroblasts were generated as described in this article (PMID 28232471). More information in Methods Section.

Authentication

The genotype of each murine organoid line was validated by Transnetyx real-time PCR to ensure they carry mutant alleles of Kras, Trp53 and/or Nrf2, where appropriate. Patient-derived cell lines were characterized by sequencing DNA to confirm that they harbor loci representative of human PDA. Cancer associated fibroblasts (CAFs) were validated by flow cytometry for positive expression of Fibroblast activating protein (FAP). CAFs were genotyped to ensure they do not carry mutant alleles of Kras, Tp53.

Mycoplasma contamination

Once the cells are cultured in our laboratories, they were screened monthly for mycoplasma contamination using a commercially available mycoplasma PCR detection kit (Sigma, MP0035).

Commonly misidentified lines
(See ICLAC register)

No cell lines used are listed in the database of commonly misidentified cell lines.

Animals and other organisms

Policy information about studies involving animals; ARRIVE guidelines recommended for reporting animal research

Laboratory animals

Gender balanced C57B/6J Mus musculus were used in the current study. KrasG12D;p53R172H;PdxCre (KPC) animals were enrolled into therapeutic studies when tumor volumes reach ~270mm3 by ultrasound imaging. Given the tumor latency of the KPC model, this generally corresponds to mice of 4-5 months of age. For orthotopic transplant experiments, 8 week old C57B6/J mice were purchased from Jackson Laboratory and were used.

Wild animals

This study did not involve wild animals

Field-collected samples

This study did not involve samples collected from the field

Ethics oversight

All experiments were conducted in accordance with procedures approved by the IACUC at Columbia University (AC-AAAT7469).

Note that full information on the approval of the study protocol must also be provided in the manuscript.

ChIP-seq

Data deposition

☐ Confirm that both raw and final processed data have been deposited in a public database such as GEO.

☐ Confirm that you have deposited or provided access to graph files (e.g. BED files) for the called peaks.

Data access links<br>*May remain private before publication.*	*For "Initial submission" or "Revised version" documents, provide reviewer access links. For your "Final submission" document, provide a link to the deposited data.*
Files in database submission	*Provide a list of all files available in the database submission.*
Genome browser session<br>(e.g. UCSC)	*Provide a link to an anonymized genome browser session for "Initial submission" and "Revised version" documents only, to enable peer review. Write "no longer applicable" for "Final submission" documents.*

Methodology

Replicates	*Describe the experimental replicates, specifying number, type and replicate agreement.*
Sequencing depth	*Describe the sequencing depth for each experiment, providing the total number of reads, uniquely mapped reads, length of reads and whether they were paired- or single-end.*
Antibodies	*Describe the antibodies used for the ChIP-seq experiments; as applicable, provide supplier name, catalog number, clone name, and lot number.*
Peak calling parameters	*Specify the command line program and parameters used for read mapping and peak calling, including the ChIP, control and index files used.*
Data quality	*Describe the methods used to ensure data quality in full detail, including how many peaks are at FDR 5% and above 5-fold enrichment.*
Software	*Describe the software used to collect and analyze the ChIP-seq data. For custom code that has been deposited into a community repository, provide accession details.*

Flow Cytometry

Plots

Confirm that:

☒ The axis labels state the marker and fluorochrome used (e.g. CD4-FITC).

☒ The axis scales are clearly visible. Include numbers along axes only for bottom left plot of group (a 'group' is an analysis of identical markers).

☐ All plots are contour plots with outliers or pseudocolor plots.

☐ A numerical value for number of cells or percentage (with statistics) is provided.

Methodology

Sample preparation	Sample preparation listed in Methods
Instrument	FACS Fortessa
Software	FACSDiva for collection and FlowJo (version 10) for analysis
Cell population abundance	At least 10,000 events were recorded for every flow cyotmetry experiment. Live cells were gated and analyzed for the relevant marker stained. Cherry-positive murine PDA cells transduced with sgRNAs targeting the ROSA locus or against different regions of the Slc2a6 gene were sorted before plating for glucose uptake and seahorse experiments.
Gating strategy	DAPI was used to gate for viable cells. FSC-A and FSC- H were used to sort for singlets. All analyses were performed in DAPI-negative, viable cells.

☐ Tick this box to confirm that a figure exemplifying the gating strategy is provided in the Supplementary Information.

Magnetic resonance imaging

Experimental design

Design type	*Indicate task or resting state; event-related or block design.*

Design specifications | *Specify the number of blocks, trials or experimental units per session and/or subject, and specify the length of each trial or block (if trials are blocked) and interval between trials.*

Behavioral performance measures | *State number and/or type of variables recorded (e.g. correct button press, response time) and what statistics were used to establish that the subjects were performing the task as expected (e.g. mean, range, and/or standard deviation across subjects).*

Acquisition

Imaging type(s) | *Specify: functional, structural, diffusion, perfusion.*

Field strength | *Specify in Tesla*

Sequence & imaging parameters | *Specify the pulse sequence type (gradient echo, spin echo, etc.), imaging type (EPI, spiral, etc.), field of view, matrix size, slice thickness, orientation and TE/TR/flip angle.*

Area of acquisition | *State whether a whole brain scan was used OR define the area of acquisition, describing how the region was determined.*

Diffusion MRI ☐ Used ☐ Not used

Preprocessing

Preprocessing software | *Provide detail on software version and revision number and on specific parameters (model/functions, brain extraction, segmentation, smoothing kernel size, etc.).*

Normalization | *If data were normalized/standardized, describe the approach(es): specify linear or non-linear and define image types used for transformation OR indicate that data were not normalized and explain rationale for lack of normalization.*

Normalization template | *Describe the template used for normalization/transformation, specifying subject space or group standardized space (e.g. original Talairach, MNI305, ICBM152) OR indicate that the data were not normalized.*

Noise and artifact removal | *Describe your procedure(s) for artifact and structured noise removal, specifying motion parameters, tissue signals and physiological signals (heart rate, respiration).*

Volume censoring | *Define your software and/or method and criteria for volume censoring, and state the extent of such censoring.*

Statistical modeling & inference

Model type and settings | *Specify type (mass univariate, multivariate, RSA, predictive, etc.) and describe essential details of the model at the first and second levels (e.g. fixed, random or mixed effects; drift or auto-correlation).*

Effect(s) tested | *Define precise effect in terms of the task or stimulus conditions instead of psychological concepts and indicate whether ANOVA or factorial designs were used.*

Specify type of analysis: ☐ Whole brain ☐ ROI-based ☐ Both

Statistic type for inference
(See Eklund et al. 2016) | *Specify voxel-wise or cluster-wise and report all relevant parameters for cluster-wise methods.*

Correction | *Describe the type of correction and how it is obtained for multiple comparisons (e.g. FWE, FDR, permutation or Monte Carlo).*

Models & analysis

n/a	Involved in the study
☐	☐ Functional and/or effective connectivity
☐	☐ Graph analysis
☐	☐ Multivariate modeling or predictive analysis

Functional and/or effective connectivity | *Report the measures of dependence used and the model details (e.g. Pearson correlation, partial correlation, mutual information).*

Graph analysis | *Report the dependent variable and connectivity measure, specifying weighted graph or binarized graph, subject- or group-level, and the global and/or node summaries used (e.g. clustering coefficient, efficiency, etc.).*

Multivariate modeling and predictive analysis | *Specify independent variables, features extraction and dimension reduction, model, training and evaluation metrics.*
